# APE1/Ref-1 Regulates STAT3 Transcriptional Activity and APE1/Ref-1–STAT3 Dual-Targeting Effectively Inhibits Pancreatic Cancer Cell Survival

**DOI:** 10.1371/journal.pone.0047462

**Published:** 2012-10-19

**Authors:** Angelo A. Cardoso, Yanlin Jiang, Meihua Luo, April M. Reed, Safi Shahda, Ying He, Anirban Maitra, Mark R. Kelley, Melissa L. Fishel

**Affiliations:** 1 Division of Hematology/Oncology, Department of Medicine, Indiana University School of Medicine, Indianapolis, Indiana, United States of America; 2 Section of Hematology/Oncology, Department of Pediatrics, Herman B Wells Center for Pediatric Research, Indianapolis, Indiana, United States of America; 3 Department of Oncology, Johns Hopkins University School of Medicine, Baltimore, Maryland, United States of America; 4 Department of Pharmacology and Toxicology, Indiana University of School of Medicine, Indianapolis, Indiana, United States of America; 5 Department of Biochemistry and Molecular Biology, Indiana University School of Medicine, Indianapolis, Indiana, United States of America; Dresden University of Technology, Germany

## Abstract

Pancreatic cancer is a largely incurable disease, and increasing evidence supports strategies targeting multiple molecular mediators of critical functions of pancreatic ductal adenocarcinoma cells. Intracellular redox state modulates the activity of various signal transduction pathways and biological processes, including cell survival, drug resistance and responsiveness to microenvironmental factors. Recently, it has been shown that the transcription factor STAT3 is under redox control, but the mechanisms involved in its regulation are unknown. Here, we demonstrate for the first time that STAT3 DNA binding and transcriptional activity is directly regulated by the redox function of the APE1/Ref-1 endonuclease, using overexpression and redox-specific mutational strategies, and gene knockdown. Also, pharmacological blockade of APE1/Ref-1 by the redox-selective inhibitor E3330 abrogates STAT3 DNA binding. Since APE1/Ref-1 also exerts redox control on other cancer-associated transcription factors, we assessed the impact of dual-targeting of STAT3 signaling and APE1/Ref-1 redox on pancreatic cancer cell functions. We observed that disruption of APE1/Ref-1 redox activity synergizes with STAT3 blockade to potently inhibit the proliferation and viability of human PDAC cells. Mechanistically, we show that STAT3–APE1/Ref-1 dual targeting promotes marked tumor cell apoptosis, with engagement of caspase-3 signaling, which are significantly increased in comparison to the effects triggered by single target blockade. Also, we show that STAT3–APE1/Ref-1 dual blockade results in significant inhibition of tumor cell migration. Overall, this work demonstrates that the transcriptional activity of STAT3 is directly regulated by the redox function of APE1/Ref-1, and that concurrent blockade of STAT3 and APE1/Ref-1 redox synergize effectively inhibit critical PDAC cell functions.

## Introduction

Pancreatic cancer remains a largely incurable disease, with patients facing the worst 5-year survival rate of any cancer. The challenge is to identify molecular effectors that critically regulate the survival of pancreatic ductal adenocarcinoma (PDAC) cells, to devise effective molecular-targeted strategies that can prevent or minimize the selection of resistant tumor variants, and overcome the protective role of the tumor-associated fibrosis and stroma. Increasing evidence supports the need for strategies targeting multiple molecular effectors in PDAC. Thus, a strategy is to identify critical molecules that regulate multiple signaling mediators (as transcription factors) and intracellular mechanisms with direct effects on multiple pathways critical for PDAC functions.

APE1/Ref-1 (hereafter referred to as APE1) is a dual function protein, which in addition to DNA repair activity also exerts redox control of transcription factors, including NF-κB, p53, AP-1, HIF-1 and others [1], [2]. Treatment with E3330, a small molecule redox signaling inhibitor that recognizes an alternate, redox active conformation of APE1 [3] markedly inhibits the DNA binding and transcriptional activity of NF-B, AP-1, and HIF-1 [4], [5]. Functioning as a redox factor, APE1 stimulates the DNA binding activity of transcription factors by reducing cysteine residues in the DNA binding domain of the ‘target’ transcription factor. [6] While the organism possesses general reduction-oxidation systems (thioredoxin and glutaredoxin/glutathione), [7], [8] APE1 functions differently as it selectively regulates factors that directly govern critical cellular functions, including hypoxia, DNA repair, inflammation, and angiogenesis. [4], [9], [10] Our previous work established APE1 as a potential molecular target in PDAC, by demonstrating that human adenocarcinoma and peri-pancreatic metastases exhibit increased APE1 expression [11], and that blockade of APE1 redox activity delays tumor progression in xenograft models of human PDAC, including patient-derived tumor cells [4].

STAT3 is a transcription factor that regulates critical cell functions and plays important roles in several cancers [12]–[15]. STAT3 signaling has been implicated in pancreatic cancer biology, namely by mediating or regulating cell survival, tumor angiogenesis and metastasis [16]–[18]. Although STAT3 signaling can be engaged and modulated by different processes, the impact of oxidative stress and its redox status are largely unknown. A recent report demonstrated that STAT3 activity is under redox control and identified the critical oxidation-sensitive cysteines in the STAT3 DNA binding domain [19], [20]. However, the modifier of STAT3 which converts it from an oxidized into a reduced form has not been identified. APE1 physically interacts with STAT3 on the VEGF promoter [21] and enhances IL-6-induced DNA binding activity of STAT3 in HepG2 cells [22]. However, it is unknown whether APE1 is involved in the redox control of STAT3 activity, and whether the cellular redox status affects STAT3 signaling in PDAC cells.

Here, we demonstrate that APE1 redox activity regulates STAT3 DNA binding and transcriptional activity, using gene silencing, overexpression of WT or redox-defective APE1, and redox-selective pharmacological inhibition. Blockade of APE1 redox synergizes with STAT3 selective antagonists to markedly inhibit the proliferation and survival of human PDAC cells, promoting cell apoptosis. These studies identify the mechanism by which APE1 regulates STAT3 activity, and establishes the rationale for the development of APE1– STAT3 dual-targeting strategies for the treatment of PDAC.

## Results

### Redox Control of STAT3 Activity in PDAC Cells

**Figure 1:**
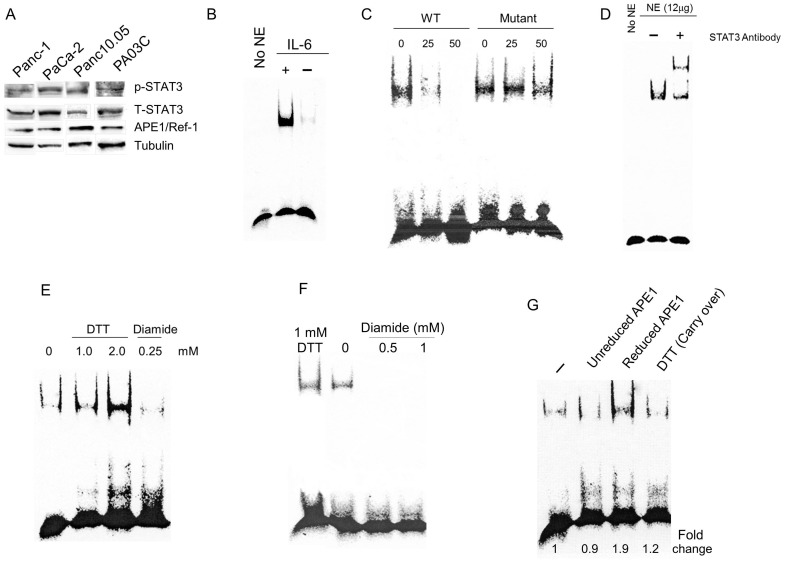
STAT3 is under APE1 redox control in PDAC cells. A) Immunoblotting for STAT3 (pY705) in PDAC cells; total STAT3 levels shown in middle panel. APE1 protein levels are shown for the patient-derived lines and tubulin is the loading control. B) STAT3 EMSA assay in PaCa-2 cells stimulated with IL-6, NE  =  nuclear extract; C) Competitive EMSA on STAT3 using nuclear extract from PaCa-2 cells and either 25- or 50-fold cold probe (WT or STAT3 binding-defective mutant); D) Supershift of STAT3 DNA binding using nuclear extract; E) STAT3 EMSA assay following treatment with DTT and diamide. F) STAT3 EMSA assay following treatment with DTT or increasing concentrations of diamide, as indicated. G) STAT3 EMSA assay with reduced APE1. DTT (Carry-over) reflects 0.04 mM DTT as used in the reaction after reducing the APE1 protein. For all EMSA assays, nuclear extract from PaCa-2 cells was treated with IL-6 (50 ng/mL) for 2 hrs in 2% FBS, unless otherwise indicated.

Although STAT3 DNA binding is reportedly under redox control [20], the molecular mechanism mediating this regulation is unknown. Here, we investigated whether APE1 regulates the DNA binding and transcriptional activities of STAT3 in PDAC. We confirmed activation of STAT3 signaling using immunoblotting and EMSA (Figure 1A, B). Both patient-derived and immortalized cell lines express APE1and exhibit STAT3 phosphorylation (residue Y705) and DNA binding; as expected, STAT3 DNA binding is enhanced following stimulation with IL-6. To confirm the specificity of STAT3 DNA binding, we performed EMSA competition assays using cold DNA probes (WT or mutant, STAT3-binding-defective sequences). As shown in Figure 1C, a dose-dependent decrease in STAT3 DNA binding was observed using the WT competitor probe which wasn’t observed using the mutant probe. Specificity of this interaction was also demonstrated by a supershift band observed using a STAT3-specific antibody (Figure 1D).

We investigated the effects of oxidizing and reducing conditions on STAT3 binding to DNA as well as its putative regulation by APE1, using PDAC nuclear extracts and treatment with diamide or DTT. Oxidizing conditions (diamide) abrogate the binding of STAT3 to DNA (Figure 1E, F); in contrast, nuclear extracts treated with the reducing agent DTT showed enhanced STAT3 DNA binding in a dose-dependent manner (Figure 1E; 1.4 to 2-fold increase). Since the redox status of STAT3 affects its DNA binding activity, we then evaluated whether the redox function of APE1 modulates its DNA binding. Addition of reduced APE1 protein to PaCa-2 nuclear extracts increased STAT3 DNA binding 1.8-fold (Figure 1G). As controls, we demonstrated that unreduced APE1 and carry-over DTT from the reduction of APE1 (0.04 mM) do not stimulate STAT3 DNA binding. These studies indicate that STAT3 signaling is activated in PDAC, and that STAT3 binding to DNA is redox sensitive and is regulated by APE1.

### STAT3 Transcriptional Activity is Increased by APE1 Overexpression and is Inhibited by APE1 Knockdown

**Figure 2:**
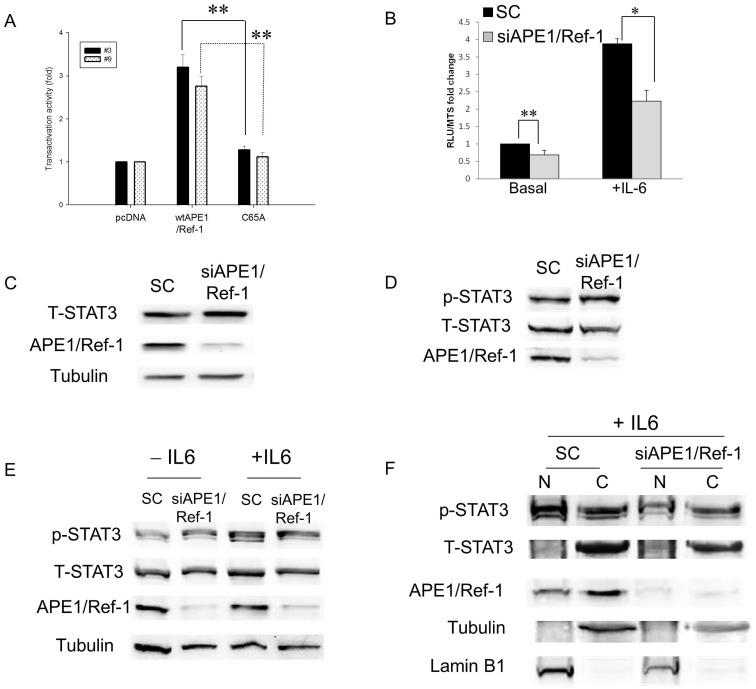
Effects of modulation of APE1 expression on STAT3 transactivation in PDAC cells. A) Panc-1 cells transiently transfected with pcDNA3, pcDNA3-wt-APE1, or pcDNA3-C65A-APE1 were assayed for STAT3 activity via luciferase reporter assay at 30 hr post-transfection. **p<0.01, n = 3 for each clone comparing wt to C65A. B) STAT3 Reporter assay of Panc-1 cells transfected with scrambled or APE1 siRNA (50 nM) and stimulated with IL-6 (50 ng/mL, 6 hr). * p<0.05, **p<0.01, n = 6 comparing SC to siAPE1. C) Western blot of total STAT3 protein levels following APE1 knockdown in PaCa-2 cells. Tubulin was used as a loading control. D) p-STAT3 (Y705) levels following APE1 knockdown in PaCa-2 cells. E) Immunoblots of whole cell extracts showing that stimulation with IL-6 (50 ng/mL, 3 hrs) increases p-STAT3 levels in both SC- and siAPE1- treated cells but does not change the levels of total STAT3 protein. F) Western blot of fractionated PaCa-2 cells stimulated with IL-6 and transfected with siRNA: nuclear (N) and cytoplasmic (C) fractions. Tubulin was utilized as a loading control for cytoplasm and Lamin B1 for nuclear.

Due to the multifunctional nature of APE1 with both DNA repair and redox activities, we performed experiments to further demonstrate that APE1 and its redox function is required for STAT3 DNA binding and activity. Using lentiviral transcriptional reporter vector, pGreenFire-STAT3-Luc (pGF-STAT3-Luc) and pGreenFire-mCMV (negative control), from System Biosciences Inc. (Mountainview, CA), we generated stably expressing reporter cell lines to assay for STAT3 activity, similar to constructs used in previous studies with NF-κB, AP-1, and HIF-1 [4]. Panc-1 cells were transduced with pGF-STAT3-Luc, and single colonies were screened for basal STAT3 and IL-6-stimulated STAT3 activity. PDAC cells expressing STAT3-driven luciferase were transfected to transiently overexpress wtAPE1 or the redox-defective mutant C65A-APE1 (C65A). Overexpression of wtAPE1 stimulates the activity of STAT3 in both clones (∼3-fold), an effect that was not seen in cells expressing the redox-dead APE1 mutant (Figure 2A). In overexpression experiments in Figure 2A, single colonies were assayed for luciferase activity after transient transfection with pcDNA, pcDNA-wt-APE1, and pcDNA-C65A-APE1, and the normalization was done using Renilla as a transfection control.

### Knockdown of APE1 does not Affect Total STAT3 Protein Levels, the STAT3 Phosphorylation or Nuclear Translocation

We then assessed the impact of APE1 knockdown on STAT3 activity in PDAC cells. As above, single colonies of pGF-STAT3-Luc were transiently transfected with APE1 siRNA for the experiments shown in Figure 2B. Results from pGF-STAT3-Luc clones #3 and #9 were pooled in experiments shown in Figure 2B with representative experiments for each clone shown in Figure S1A, B. Basal levels of STAT3 activity are significantly diminished when APE1 expression is knocked down using transient, specific siRNA (Figure 2B; p<0.001). The decrease in STAT3 activity by APE1 knockdown was more pronounced in cells stimulated with IL-6, further supporting a regulatory role for APE1 in the DNA binding activity of STAT3 (Figure 2B, p<0.05; and Figures S1A, B). The effects on STAT3 transcriptional activity when APE1 levels are decreased are not due to an increase in total STAT3 protein as shown by immunoblotting for total STAT3 protein (Figure 2C and quantitation of blots in Figure S1C) and by qPCR for STAT3 mRNA (Figure S1D). We also performed studies to show that the impact of APE1 on STAT3 signaling was restricted to the regulation of its DNA binding activity, and did not affect levels of STAT3 phosphorylation (Figure 2D and quantitation in Figure S1E). Furthermore, stimulation with IL-6 under conditions of APE1 knockdown did not affect total STAT3 protein levels (Figure 2E). As expected, stimulation with IL-6 increases the levels of p-STAT3, and this regulation of STAT3 activity is not dependent upon APE1 protein levels.

Next, we investigated the nuclear translocation of STAT3 by probing lysates from nuclei and cytoplasm of PaCa-2 cells transfected with APE1 siRNA or SC control; total STAT3 and phospho-STAT3 (Y705) were analyzed, with tubulin and Lamin B used as cytoplasmic and nuclear controls, respectively. As expected, stimulation with IL-6 resulted in marked increase in the levels of p-STAT3 (Fig. 2E, F). Normalization of p-STAT3 to Lamin B levels showed that there were no significant differences in nuclear p-STAT3 in the APE1-silenced versus SC control (0.83 fold change). This finding was confirmed both in whole cell extracts (Figure 2 D, E) and in nuclear extracts (Figure 2F). These observations further indicate that APE1 regulates STAT3 DNA binding without affecting other mechanisms of regulation, i.e. phosphorylation, nuclear translocation, or amount of total STAT3 protein under basal or IL-6-induced conditions.

### APE1 Redox Inhibitor, E3330 Inhibits STAT3 Transcriptional Activity

**Figure 3:**
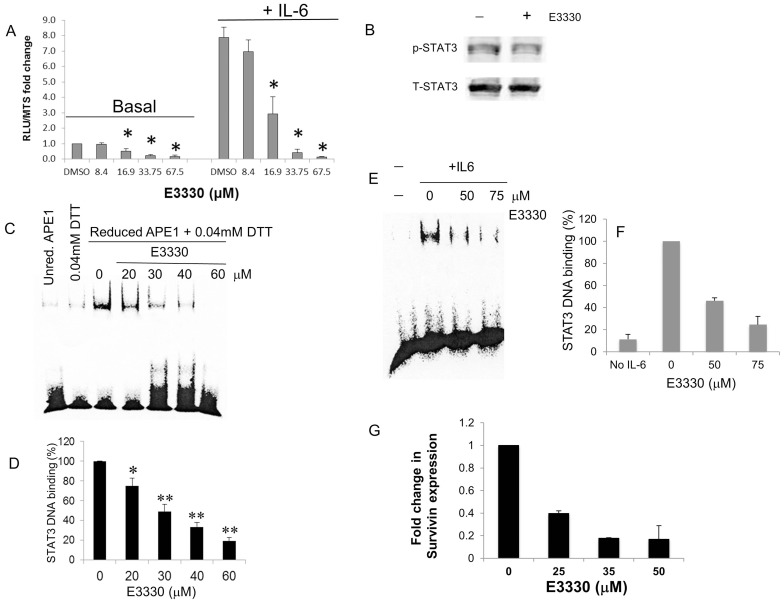
STAT3 activity following treatment of PDAC cells with APE1 redox inhibitor, E3330. A) STAT3 reporter assay in Panc-1 cells following treatment with E3330 (24 h) and stimulation with IL-6 (6 h). Doses were based on survival data from previously published data [4]. * p<0.05 t test, n = 6 comparing DMSO control to drug-treated samples. B) Representative Western blot of PaCa-2 cells treated with E3330 (50 µM, 24 hr). C) Addition of E3330 to the EMSA reaction inhibits the STAT3 DNA binding of nuclear extracts stimulated with IL-6. Representative EMSA blot shown with quantitation of three independent experiments in D. * p<0.05, ** p<0.01, compared to DMSO control, using Student’s t test. E) STAT3 DNA binding via EMSA assay following treatment of PaCa-2 cells with E3330 (2 h) and then stimulated with IL-6 (50 ng/mL, 2 h) with quantitation in (F) where intensity of binding was compared to amount of STAT3 DNA binding with IL-6 stimulation; G) qPCR analysis of Survivin expression following E3330 treatment for 24 h in patient-derived cells, Pa02C. Shown is data from 2–3 individual experiments (avg±SE). Samples were run in triplicate and normalized to DMSO vehicle control.

To more specifically address the role of the redox function of APE1 on STAT3 transcriptional activity, we performed experiments using the small molecule E3330, which selectively inhibits APE1 redox activity without affecting its endonuclease function. [3], [5] E3330 markedly inhibits STAT3 activity both in stable lines (Figure 3A; Figures S2A,B) as well as in transient luciferase assays (Figure S2C). Basal STAT3 activity was significantly inhibited by E3330 (Figure 3A; p<0.05) in a dose-dependent manner. Also, E3330 treatment significantly inhibited the STAT3 activity induced by IL-6 stimulation of PDAC cells (Figure 3A; p<0.05), which was abrogated at higher E3330 doses. This inhibition of STAT3 activity was not due to a decrease in total STAT3 protein levels or a reduction in p-STAT3 levels. Results from immunoblotting of whole cell lysates demonstrated that the amount of STAT3 protein and phosphorylated STAT3 protein (0.95-fold compared to DMSO control) after treatment with APE1 redox inhibitor, E3330 in PaCa-2 cells was not significantly changed (Figure 3B).

To further confirm the inhibition of STAT3 DNA binding by APE1 redox blockade, nuclear extracts from PaCa-2 cells were treated with E3330 and analyzed by EMSA for STAT3 binding; a dose-dependent decrease in STAT3 binding was observed (Figure 3C), with an IC_50_ for E3330 around 30 µM (Figure 3D). We also treated PaCa-2 cells with E3330 and then stimulated STAT3 DNA binding with IL-6 and assayed for STAT3 DNA binding using EMSA assay. As shown in Figure 3E and F, IL-6-induced STAT3 activity is inhibited dramatically following E3330 treatment in cells. Further supporting a decrease in STAT3 DNA binding following inhibition of APE1 redox activity, expression of STAT3 target gene, Survivin is decreased in a dose-dependent manner both in patient-derived cells (Figure 3G) and in PaCa-2 cells (Figure S2D). These studies demonstrate that manipulation of APE1 expression and disruption of its redox function markedly affects STAT3 DNA binding and transcriptional activity.

### Pharmacologic Blockade of APE1 and STAT3 Results in Synergistic Effects on Human PDAC Cells

**Figure 4:**
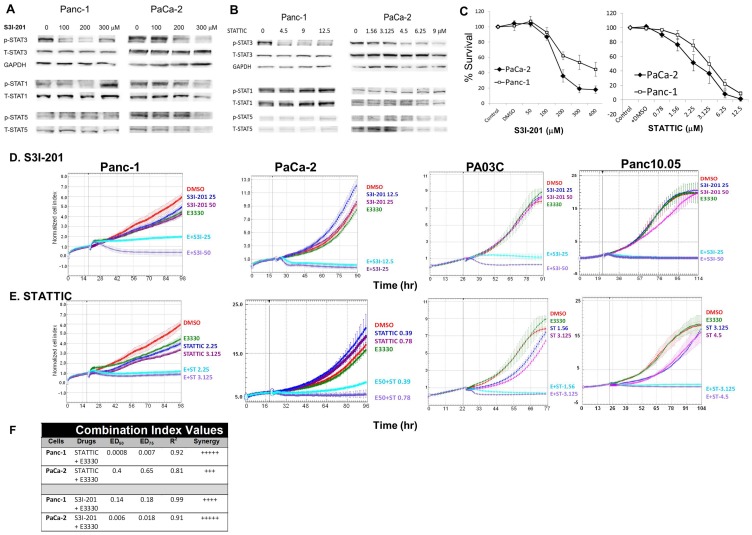
STAT3– APE1 dual targeting effectively inhibits PDAC cell proliferation. Immunoblotting of STAT proteins, p-STAT3(Y705), p-STAT1(Y701), and p-STAT5(Y694) in PDAC cells treated with S3I-201 (A) or STATTIC (B). DMSO was used as vehicle control and is included in the lane without STAT3 inhibitor. Panc-1 cells were treated with S3I-201 for 30 hr and STATTIC for 30 min. PaCa-2 cells were treated with S3I-201 and STATTIC for 24 hr. C) Dose response in PaCa-2 and Panc-1 cells following 72 hr treatment with S3I-201 or STATTIC via MTS assay. D, E) Cell proliferation after E3330 (50 µM for all lines, except 40 µM for Panc10.05) and S3I-201 or STATTIC treatment. Assays were performed in duplicate, representative experiment, from 2–4 independent experiments. F) Combination index (CI) values calculated with Calcusyn for combination of APE1 and STAT3 inhibitors at their ED_50_s in the MTS assay. CI values of <0.1, very strong synergy; 0.1–0.3: strong synergy; and 0.3–0.7: synergy.

First we show that treatment with two previously described STAT3 antagonists, STATTIC [23] and S3I-201 [24] inhibited phosphorylation of STAT3 (Figure 4 A,B) as well as cellular proliferation in PDAC cells (Figure 4C). Statistically significant inhibition of phosphorylation of Y705 is observed at doses of S3I-201 greater than 100 µM and with doses of STATTIC greater than 3.125 µM, as assessed by densitometry (p<0.05). At the inhibitory doses tested, these compounds did not affect the phosphorylation levels of STAT1 (Y701) or STAT5 (Y694) in these cells (Figure 4A,B). Functional studies using the MTS assay showed that STAT3 blockade markedly inhibits proliferation of both PDAC lines (Figure 4C), with ED_50_ of ∼2.5 µM for PaCa-2 and ∼4 µM for Panc-1 for STATTIC, and ED_50_ ∼190 µM for PaCa-2 and ∼310 µM for Panc-1 for S3I-201. For both drugs, a strong association was observed between decrease in STAT3 phosphorylation and inhibition of proliferation of PDAC cells.

In addition to regulating the transcriptional activity of STAT3, APE1 also exerts redox control of other transcription factors, which have been implicated in pancreatic cancer (such as HIF-1α and NF-κB). [4], [16], [25] We then evaluated whether the combined blockade of STAT3 and APE1 redox activity synergize to more effectively inhibit human PDAC cells. Cell survival was assessed using the xCELLigence system, which monitors real-time changes in cell adherence, morphology and viability; [4], [26] cell index (CI) was monitored over 72 h drug treatment. E3330 was used at doses that effectively inhibit APE1 redox activity, and STAT3 inhibitors at doses that were utilized in the MTS-based assay for cytotoxicity (Figure 4C) and that inhibited STAT3 phosphorylation in PDAC cells (Figure 4A,B and Ref. [24]). As single agents, minimal inhibition of PDAC cells was observed with E3330 or at the doses of STATTIC or S3I-201 used (Figure 4D,E). We observed that APE1 redox inhibition by E3330 synergizes with STAT3 blockade by STATTIC or S3I-201, resulting in potent inhibition of Panc-1 and PaCa-2 cells, but also of the primary Pa03C and Panc10.05 cells (Figure 4D,E). The potentiation of the inhibitory effects seen with this dual targeting, at sub-optimal doses of these agents, was observed both using xCELLigence (Figure 4) and MTS assays (Figure S3). Interestingly, in the patient-derived cells Pa03C and Panc10.05, this enhancement was observed at lower doses of E3330 than those observed with the established cells lines (data not shown). Blockade of signaling through both APE1 redox activity and STAT3 has dramatic effects on cell survival. To demonstrate that this effect was not due to a general redox phenomenon, we also combined STAT3 inhibitors with thioredoxin inhibitor, PX-12 [27]–[29]. Addition of STAT3 inhibitor S3I-201 to PX-12 treatment did not significantly enhance the effects of PX-12 (Table S1; assessed by comparison of ED_25_, ED_50_, and ED_75_).

To quantify potential drug synergisms, the Chou-Talalay method was used with increasing dose combinations of one STAT3 antagonist plus the APE1 inhibitor E3330. The ED_50_ for the three compounds was determined as shown above, using MTS assays (Figure 4 and Ref. [4]). Panc-1 or PaCa-2 cells were treated with two-drug combinations at fixed ratio of doses corresponding to 0.125, 0.25, 0.5, 0.75, 1, 1.125, and 1.25-fold of their individual ED_50_ values. As shown in Figure 4F, potent synergisms were observed with combination index values significantly <1 for ED_50_ and ED_75_ in both cell lines. Overall, these studies show that the simultaneous targeting of APE1 redox and STAT3 signaling synergize to markedly impair the survival of human pancreatic cancer cells, even at sub-optimal doses of the individual agents.

### Dual Targeting of APE1 Redox Activity and STAT3 Triggers Increase Apoptotic Effects in PDAC Cells

**Figure 5:**
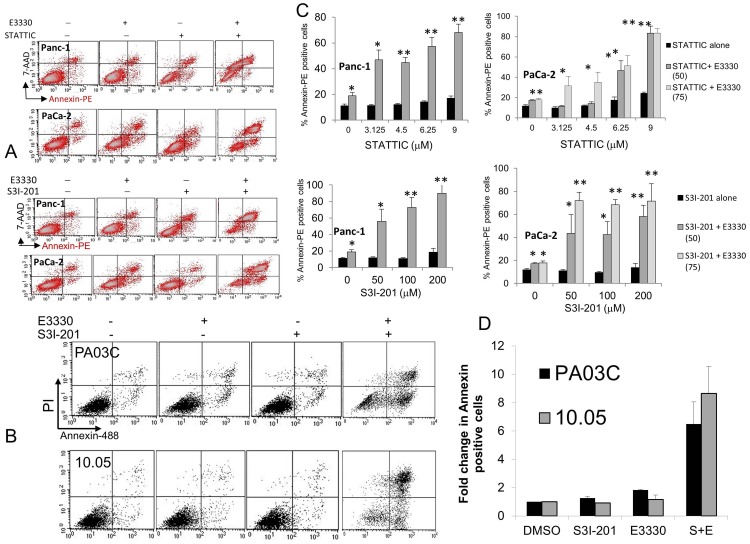
Effect on apoptosis of inhibition of APE1 in combination with STAT3 inhibitors in Panc-1, PaCa-2, and the patient-derived Pa03C and Panc10.05 cells. Inhibitors were added simultaneously (E3330 40, 50, or 75 µM), and apoptosis was determined using Annexin-V (x-axis) and 7-AAD or PI (y-axis) staining after 24 hr. Representative dot plots are shown in A and B with high dose of STAT3 inhibitor for Panc-1 and PaCa-2 and 50µMS3I-201 for the patient-derived cells. Graphical representation represents >3 independent experiments; *, p<0.05, **, p<0.01 all compared to STAT3 inhibitor alone at corresponding dose (A, C) or 2 independent experiments for (B, D).

Subsequently, we performed mechanistic studies to examine whether these synergistic effects involve increased apoptotic cell death and engagement of the Caspase pathway. First, we used the Annexin-PE/7-AAD assay to assess cell death and viability in PDAC cells treated with the drug combinations indicated (Figure 5A,C). Panels A and B depict representative plots of the flow cytometry analyses, and show that the combination of E3330 with a STAT3 inhibitor markedly increase the frequency of Annexin-positive, non-viable cells; comparable results were seen for patient-derived cells (Figure 5B, D). Analyses of data from 4 to 6 individual experiments demonstrate that the combination of E3330 with STATTIC or with S3I-201 significantly increases pancreatic cancer cell death (Figure 5B). Furthermore, similar results are observed in the patient-derived cells in which a ∼6-8-fold increase in cell death is observed with the combined targeting of APE1 and STAT3 (Figure 5B,D).

**Figure 6:**
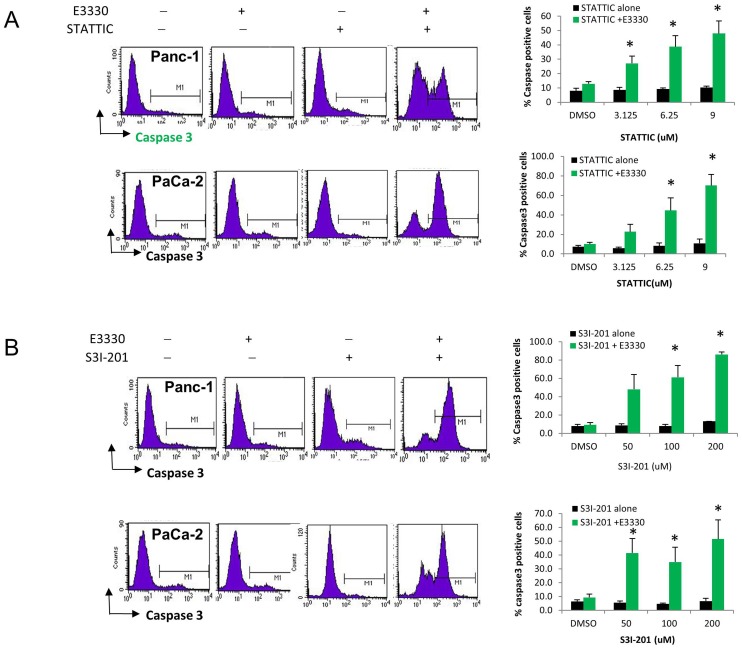
Dual STAT3– APE1 targeting activates Caspase 3 in PDAC cells. Panc-1 and PaCa-2 cells were treated with E3330 (50, 75 µM) and increasing amounts of STATTIC (A) or S3I-201 (B) and assayed for caspase 3 activity. The graphs represent >3 independent experiments; *, p<0.05, all compared to STAT3 inhibitor alone at corresponding dose.

To evaluate the potential involvement of caspase-dependent cell death, we performed experiments measuring the activation of caspase 3 in PDAC cells treated with the same drug combinations, using a FITC-conjugated caspase-3 inhibitor (FITC-DEVD-FMK) and flow cytometry. As shown in Figure 6, although single agents did not induce marked caspase 3 activation, the combinatory effects of APE1 redox inhibition and STAT3 blockade resulted in substantial activation of caspase 3, in both PDAC cell lines (Figure 6A,B, representative flow histograms in left panels). These effects were statistically significant (Figure 6B; right panels) when E3330 was combined with STATTIC (A, p<0.05) or with S3I-201 (B, p<0.05). Overall, these results show that by combining sub-optimal doses of individual selective agents, the simultaneous targeting of APE1 redox and STAT3 signaling results in significant cell death of PDAC cells, with the engagement of caspase 3 pro-apoptotic effects.

### Dual Targeting of APE1 Redox and STAT3 Signaling Significantly Inhibits the Migration of PDAC Cells

**Figure 7:**
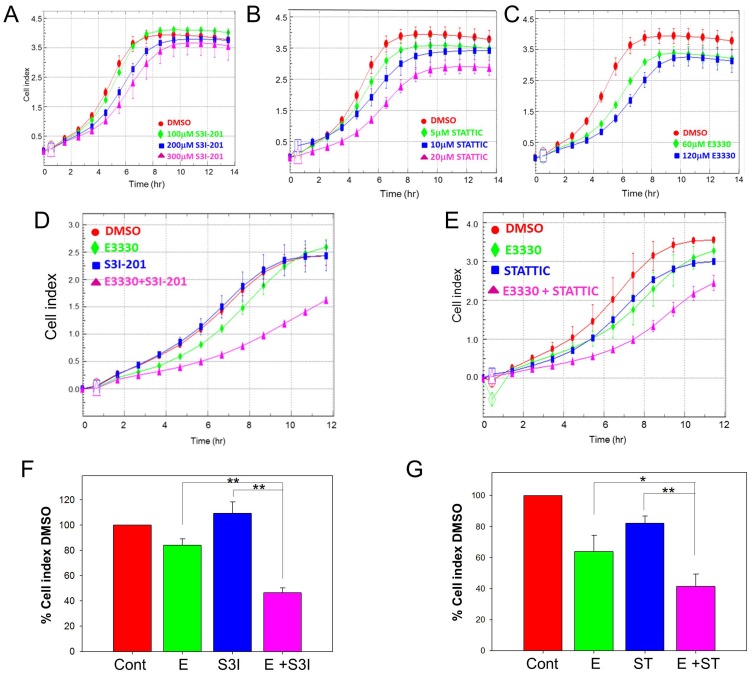
STAT3– APE1 dual targeting inhibits PDAC cell migration. Migration assayed via xCELLigence system, treatment with increasing amounts of S3I-201 (A), STATTIC (B), or E3330 (C). Combination of E3330 (75 µM) with STAT3 inhibitor S3I-201 (D), 50 µM or STATTIC (E), 10 µM dramatically reduces the cell’s migratory ability. Quantitation of three individual experiments at 8 hr is shown in F and G. * p<0.05, ** p<0.01 using paired t test comparing inhibitor alone versus combination treatment.

Finally, since cell migration represents an important step in the progression and dissemination of pancreatic cancer, we evaluated the effects of APE1 and STAT3 blockade on the response of PDAC cells to chemotactic stimuli, using the xCELLigence system. Serum-deprived Panc-1 cells were placed in upper chamber of a CIM plate, and stimulated with media supplemented with serum, which provides chemotactic stimuli (lower chamber). As expected, serum-starved Panc-1 cells do not migrate in the absence of chemotactic stimuli, and fibronectin coating helps the attachment of migrating cells (Figure S4A). Treatment of Panc-1 cells with E3330, STATTIC or S3I-201 as single agents resulted in limited inhibition of Panc-1 cell migration, even at relatively high doses of these compounds (Figure 7). However, combination of E3330 with S3I-201 (50 µM and 100 µM, Fig. S4D) or with STATTIC resulted in markedly decreased cell migration (Figure 7D,E), with both S3I-201/E3330 and STATTIC/E3330 combinations resulting in significant inhibitory effects in Panc-1 cell migration (Figure 7F, Figure S4D, p<0.01; Figure 6G, p<0.05 in comparison to E3330 alone and p<0.01 in comparison to STATTIC alone). Importantly, these drugs or combinations showed no significant effects on the viability of Panc-1 cells at the timepoints and doses assessed in the migration assays (Figure S4 B,C). These results indicate that concurrent blockade of APE1 redox and STAT3 signaling cooperates also to inhibit the migration properties of PDAC cells.

## Discussion

In this report, we demonstrate for the first time that STAT3 DNA binding is under redox control, which is mediated by the redox activity of APE1. Previous studies demonstrated that oxidation of critical cysteines residues in STAT3 protein through peroxide treatment could decrease STAT3 (but not STAT1) DNA binding and transcriptional activity [20]. Using nuclear extracts from PDAC cells, we systematically characterized the effects of reducing and oxidizing conditions on STAT3 DNA binding. Clearly, STAT3 binds to DNA more effectively when is reduced, and the redox activity of APE1 is capable of stimulating STAT3 DNA binding. Furthermore, manipulation of APE1 levels affects the DNA binding activity of STAT3. The concurrent blockade of STAT3 and APE1 redox activity acts synergistically to disrupt PDAC cell viability as well as their migration properties.

This is the first demonstration also that APE1 regulates the STAT3 DNA binding and transcriptional activity in PDAC cells. This finding points to the interaction of APE1 and STAT3 as part of the survival signaling in PDAC rather than a non-specific effect of general redox regulators. Overexpression of a redox-deficient APE1 protein failed to promote STAT3 transactivation in PDAC cells, and blockade of the redox function of APE1 via E3330 dramatically inhibited the transcriptional activity of STAT3, both basal levels as well as IL-6-induced activity. However, knockdown of APE1 decreases STAT3 activity without affecting its phosphorylation or nuclear translocation. Taken together, these data support the hypothesis that APE1 directly controls the DNA binding activity of STAT3. With the high levels of APE1 expression in PDAC tumors (Figure 1A, Ref. [11]), stimulation of STAT3 signaling through APE1’s redox activity may contribute to the threshold of STAT3 activity in the tumor leading to a more aggressive phenotype. Increase in STAT3 signal fitness through oncogenic events and/or tumor-associated extrinsic signals such as IL-6 may be partially driven through APE1.

Within the STAT family of transcription factors, STAT3 is the first STAT member to be shown to be regulated by APE1. Work by Li et al elegantly demonstrated using chimera STAT1/STAT3 proteins that STAT3, but not STAT1, is under redox control. [20] ROS can activate STAT signaling and anti-oxidants can inhibit this activation. [30] We have previously shown that knocking down of APE1 in PDAC cells does not increase ROS levels [11], and therefore its effects on STAT3 DNA-binding activity here reported cannot be attributed to changes in ROS activity. An understanding of whether other STAT family members are regulated by APE1 is important due to their diverse roles in cellular function including cytokine and growth factor signaling, differentiation, inflammation, and senescence [31]–[33], and are part of ongoing studies.

**Figure 8:**
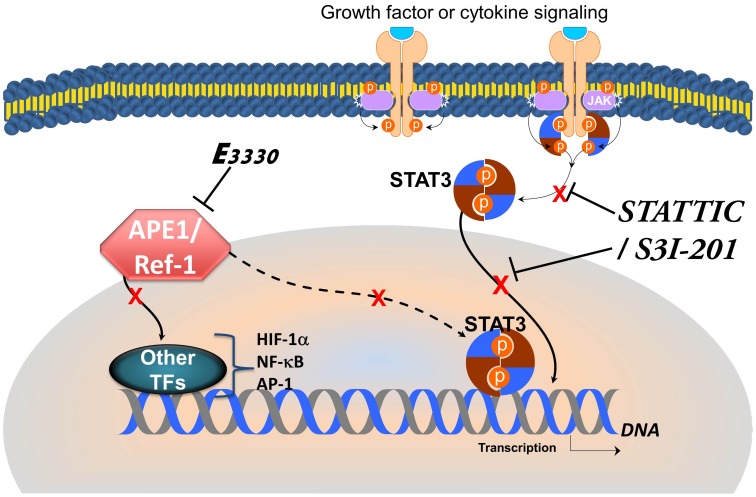
Model showing inhibition of STAT3 phosphorylation and nuclear translocation via S3I-201 or STATTIC as well as inhibition of STAT3 DNA binding via APE1 disruption. TF  =  Transcription factor.

An exciting finding in this study is the dramatic synergy seen between APE1 and STAT3 blockade in patient-derived pancreatic cancer cells. Our study demonstrates the importance of APE1 redox function in regulating STAT3 DNA binding and transcriptional activity (Figure 8). However, APE1 does not regulate only the DNA binding of STAT3, but also controls the activity of NF-κB, AP-1, and HIF-1α [1], [4]. STAT3, NF-κB, and HIF-1 signaling contribute to the crosstalk between the tumor and the tumor microenvironment (TME) [14], [34]–[36]. APE1 also plays an important role in signaling within the TME through the regulation of these transcription factors. Therefore, using inhibitors that target two critical proteins, APE1 and STAT3, we can potentially disable multiple key pathways in PDAC cell survival, and to disrupt the integration of signals between tumor and the microenvironment [37]. This is particularly important in PDAC as the disease frequently presents with metastatic disease. Our approach is consistent with the idea of synthetic lethality in which the pairing of two hits is sufficient to more effectively trigger cancer cell death, markedly improving the efficacy of single-target agents [38]. Both of these proteins are upregulated in cancer and contribute to the resistance of the disease, leaving cancer cells “addicted” to their effector functions. Dual targeting of STAT3 and APE1 is not only efficacious in PDAC; we also observed significant synergy in glioblastoma cells (Fishel, Kelley, manuscript in preparation). This is especially relevant in brain tumors, where STAT3 is important in the mesenchymal transformation [39]. As next steps, we will evaluate STAT3 and APE1 as molecular targets in xenograft models of human pancreatic cancer. A STAT3-selective inhibitor is now in a Phase I clinical trial for solid tumors and preclinical work with E3330 demonstrates its efficacy against patient-derived PDAC xenografts [4]. These studies will set the framework for a future clinical study using this dual-targeting STAT3– APE1 strategy.

The current standard of care for pancreatic cancer (debulking surgery coupled with chemotherapy and/or radiation) is largely palliative rather than curative, with rare cases of long-term regression. The effective targeting of PDAC cells remains a major clinical challenge. Monotherapies are largely ineffective, therefore one approach would be to develop two- or multi-hit approaches [38] targeting signaling pathways that critically regulate PDAC survival. Strategies, such as the one described here, involving synthetic lethality, the targeting of critical transcriptional programs, and molecular effectors of the crosstalk between the tumor and TME, may offer the most promise for clinical utility against this dreaded disease. Studies in animal models of PDAC confirm that dual- or multi-targeting approaches can increase anti-tumor responses [40], [41]. APE1 and STAT3 are upregulated and play important roles in cancer, suggesting that tumor cells may be ‘addicted’ to their effector functions. In addition to biochemical and molecular data linking APE1 and STAT3 in PDAC cells, our studies reveal potent anti-tumor synergism of the combination of APE1 redox inhibition and STAT3 blockade. Therefore, we believe that targeting of the APE1– STAT3 molecular axis has great clinical potential as a novel approach to impair multiple PDAC cell survival mechanisms.

## Materials and Methods

### Cell Lines and Patient-derived PDAC Cells

Panc-1 and PaCa-2 were purchased from and authenticated by ATCC (Manassas, VA). Pa03C, Panc10.05, and Pa02C were obtained from Dr. Anirban Maitra at The Johns Hopkins University. [42] All cells were maintained at 37°C in 5% CO_2_ and grown in DMEM (Invitrogen; Carlsbad, CA) with 10% Serum (Hyclone; Logan, UT).

### Inhibitors

E3330 was synthesized as previously described [3], [43], and STAT3 selective inhibitors S3I-201 [24] and STATTIC [23] were purchased from Calbiochem.

### qRT-PCR Reactions

qRT-PCR was used to measure the mRNA expression levels of STAT3 and STAT3 downstream target, survivin gene. PaCa-2 and patient-derived lines were treated with increasing amounts of E3330 for 24-h in media containing 1–2% serum, and total RNA was extracted from cells using the Qiagen RNeasy Mini kit (Valencia, CA) according to the manufacturer’s instructions. The extracted RNA was quantified by a Qubit fluorometer (Invitrogen Corp, Carlsbad, CA). First-strand cDNA was obtained from RNA using random hexamers and MultiScribe reverse transcriptase (Applied Biosystems, Foster City, CA). Quantitative PCR was performed using Taqman Gene Expression assays and Universal PCR master mix (Applied Biosystems) in a 7900HT Sequence detection system (Applied Biosystems). The relative quantitative mRNA level was determined using the comparative C_t_ method using Actin (PaCa-2) or ribosomal protein large, P0 (RPLP0, patient lines) as the reference gene. [4] The primers for STAT3, survivin, Actin, and RPLP0 are commercially available (Applied Biosystems). Experiments were performed in triplicate for each sample.

### Survival, Proliferation, and Synergy Studies

The proliferative capacity of PDAC cells was assessed using the xCELLigence system (Roche Applied Science, Indianapolis IN) [4], [26] as well as MTS assay as previously described [4]. Combination Index Calculations. PDAC cells were seeded into 96-well plates as described previously, and ED_50_s of E3330, S3I-201, or STATTIC on growth were determined using the MTS assay. Drug interaction was evaluated using CalcuSyn software (Biosoft, Ferguson, MO), which is based on the Chou-Talalay method [44].

### Transfection of PDAC Cells with APE1 and Scrambled siRNA

All siRNA transfections were performed as previously described [10], [43], [45], [46]. STAT3 luciferase assays were conducted on day 3 following knockdown [11].

### Western Blot Analysis

For whole cell lysates, cells were harvested, lysed in RIPA buffer (Santa Cruz Biotechnology; Santa Cruz, CA), and protein was quantified and electrophoresed. Nuclear and cytoplasmic extracts were isolated as in [47]. Immunoblotting was performed using the following antibodies: APE1 (Novus Biologicals; Littleton, CO), STAT1, STAT3, STAT5, p-STAT1(Y701), p-STAT3(Y705), p-STAT5 (Y694) (Cell Signaling; Danvers, MA), and tubulin (Sigma Aldrich) or GAPDH (Santa Cruz).

### Electrophoretic Mobility Shift Assay (EMSA)

EMSA were performed as previously described [48] with the following modifications. For super-shift assay, 6 µg STAT3 antibody (Santa Cruz Biotechnology, Inc, Santa Cruz, CA) was pre-incubated with 15 µg nuclear extract from PaCa-2 cells (treated with 50 ng/ml IL-6 for 2 hrs in 2% serum), followed by 1 µg/ul poly(dI-dC) • poly(dI-dC) (Amersham Biosciences, Piscataway, NJ) and 0.1 pmol 5′HEX-labeled double-stranded oligonucleotide DNA (Midland Certified Reagent Company, Midland, TX) containing the STAT3 direct repeat consensus sequence (5′- GAT CCT TCT GGG AAT TCC TAG ATC-3′) for 15 min. [49] For the experiment of APE1/STAT3 interaction, purified APE1 protein was reduced with 2 mM DTT for 10 min and diluted to a final concentration of 4 µg with 0.4 mM DTT in PBS. Reduced APE1 was added to 15 µg nuclear extract as above. The final concentration of DTT in redox reactions was 0.04 mM. For EMSA with E3330 treatment on nuclear extracts, E3330 was pre-incubated with purified, reduced APE1 in EMSA reaction buffer for 30 min, followed by addition of 3 µg nuclear extract.

### Apoptosis and Caspase 3 Activation Assays

Apoptosis was assayed 24 h after inhibitor treatment in the conditions indicated, using Annexin-V/7-AAD [4], [50] by flow cytometry. Activation of Caspase 3 was assessed using the FITC-conjugated DEVD-FMK inhibitor in permeabilized PDAC cells, by flow cytometry.

### Stable Cell Lines for Reporter Assays

Lentiviral transcriptional reporter vectors pGreenFire- STAT3 and the control pGreenFire-mCMV, (System Biosciences Inc., Mountainview, CA) were used to transduce Panc-1 cells, as previously reported. [4] For overexpression experiments, colonies were cotransfected with control pcDNA vector, pcDNA-wtAPE1 or pcDNA-C65A-APE1 and *Renilla* luciferase vector, pRL-CMV (Promega Corp., Madison, WI), in a 1∶10 ratio using Lipofectamine2000. *Firefly* and *Renilla* luciferase activities were assayed using the Dual Luciferase Reporter Assay System (Promega Corp., Madison, WI). Transfection experiments were performed in triplicate and repeated at least three times as independent experiments.

For STAT3 transactivation experiments with APE1 siRNA, Panc-1 colonies were transfected with siRNA and assayed for STAT3 activity. With E3330 treatment, cells were treated for 24 h in serum free media and then with IL-6 for 6 h. RLU was normalized to cell viability (MTS assay) as previously described. [4].

### Migration Assay

To assay for migration, we utilized xCELLigence DP cell invasion and migration (CIM) system. Cells were serum starved for 18 h and then seeded at 8×10^4^ in 80 µl of serum-free medium in the upper chamber with 8 µM pore size while the lower chamber contained media with 10% serum. Cells were pretreated with the inhibitors indicated for 2 h, prior to addition to the well. Cell migration and viability were monitored every hour for 14 h.

### Statistics

All data points for vehicle, E3330, STAT3 inhibitor, and combination treatments were analyzed. Statistical analyses were performed using the paired t-test, and for the apoptosis experiments, the one way ANOVA test was used (Sigma Plot software). Differences between groups were considered significant if p<0.05. For the analysis of Combination Index (CI) values using Chou-Talalay method, the Calcusyn program provided the CI values based on a Dose Effect Analysis. Curves generated using single agent or combination treatment are scored with an r value, the linear correlation coefficient. For these experiments, we required the r value to be above or equal to 0.9. If this requirement is met, CI values are generated which are quantitative measures of the degree of drug interaction based on enzyme kinetic models.

## Supporting Information

Figure S1 **STAT3 activity is inhibited by APE1 knockdown, however STAT3 mRNA and protein levels do not change.** Representative experiment of Panc-1 cells transduced with pGF-STAT3-Luc clones #3 (A) and #9 (B) following transfection with scrambled or APE1 siRNA (50 nM) and induced with IL-6 (50 ng/mL, 6 hr). C) Quantitation of Western blot of total STAT3 protein levels after APE1/Ref-1 knockdown in PaCa-2 cells. Total STAT3 levels were normalized to Tubulin. D) The amount of mRNA for STAT3 was analyzed by qPCR, using RPLP0 as the internal control for patient-derived lines (black bars) and Actin mRNA as the internal control for PaCa-2 (gray bars). For the patient-derived lines, the mRNA from three specimens was measured separately, in triplicate, and then averaged. PaCa-2 was done in three separate experiments in triplicate and the data averaged. E) Quantitation of Western blot for p-STAT3 levels following APE1 knockdown in PaCa-2 cells. p-STAT3 levels were normalized to total STAT3. Data represent average ± SD and are expressed as treated to scrambled (SC) control (n = 4–6).
(TIF)

Figure S2 **Inhibition of APE1 redox activity results in a decrease in STAT3 activity via reporter assay and target gene expression.** A, B) Representative experiment of STAT3 activity following treatment of Panc-1 clones with APE1 redox inhibitor, E3330. C) Panc-1 cells were transiently transfected with STAT3-Luc construct and cotransfected with a *Renilla* vector, pRL-TK. After 16 h, cells were treated with E3330 for 24 h, IL6 (50 ng/mL) for 6 h, and *Firefly* and *Renilla* luciferase activities were assayed using *Renilla* luciferase activity for normalization. All transfection experiments were performed in triplicate and repeated at least 4 times in independent experiments. Data are expressed as Relative Luciferase Units (RLU) normalized to DMSO, and mean ± SE are shown. Student’s *t* tests were performed; * *p*<0.05, comparing E3330 versus DMSO. D) Expression of STAT3 target gene, survivin goes down following E3330 treatment (24 hr) in PaCa-2 cells (n = 3, avg±SD) via qPCR.
(TIF)

Figure S3 **STAT3-APE1 dual targeting effectively inhibits PDAC cell proliferation.** MTS assay was used to determine cell survival. Both drugs were added and were present for 72 h. Panc-1 and PaCa-2 were treated with 50 µM E3330. DMSO was tested as vehicle control. Data shown as mean ± SE of at least four independent experiments.
(TIF)

Figure S4 **Combination of STAT3 and APE1 inhibitors inhibit PDAC cell migration.** A) Panc-1 cells were serum-starved overnight. Cells (6×10^5^) were plated in duplicate in the upper chamber CIM plates with or without FN coating. Cells were also plated in the presence and absence of FBS in the lower chamber. Readings were taken for 12 h following plating. To demonstrate that the cells plated for migration shown in Figure 7 were indeed viable and that the migration of live cells was being monitored, we tested concurrent E-plate assays (B) using the xCELLigence system or MTS assays (C). Cont  =  DMSO, E = E3330, S3I = S3I-201, ST =  STATTIC. D) Treatment with E3330 (75 µM) with STAT3 inhibitor S3I-201 (100 µM), dramatically reduces the cells’ migratory ability. Quantitation of three individual experiments at 8 hr is shown in F and G. ** p<0.01 using paired t test comparing S3I-201 alone with combination treatment.
(TIF)

Table S1 **Dual targeting of thioredoxin and STAT3 is not synergistic in PDAC cells.** ED_25_, −_50_, and −_75′_s were determined using the MTS assay.
(PPTX)
