# Supplementary figures and images for: APE1/Ref-1 Regulates STAT3 Transcriptional Activity and APE1/Ref-1–STAT3 Dual-Targeting Effectively Inhibits Pancreatic Cancer Cell Survival
Source: PLoS One. 2012 Oct 19;7(10):e47462. doi: 10.1371/journal.pone.0047462 (PMC3477158; doi:10.1371/journal.pone.0047462)

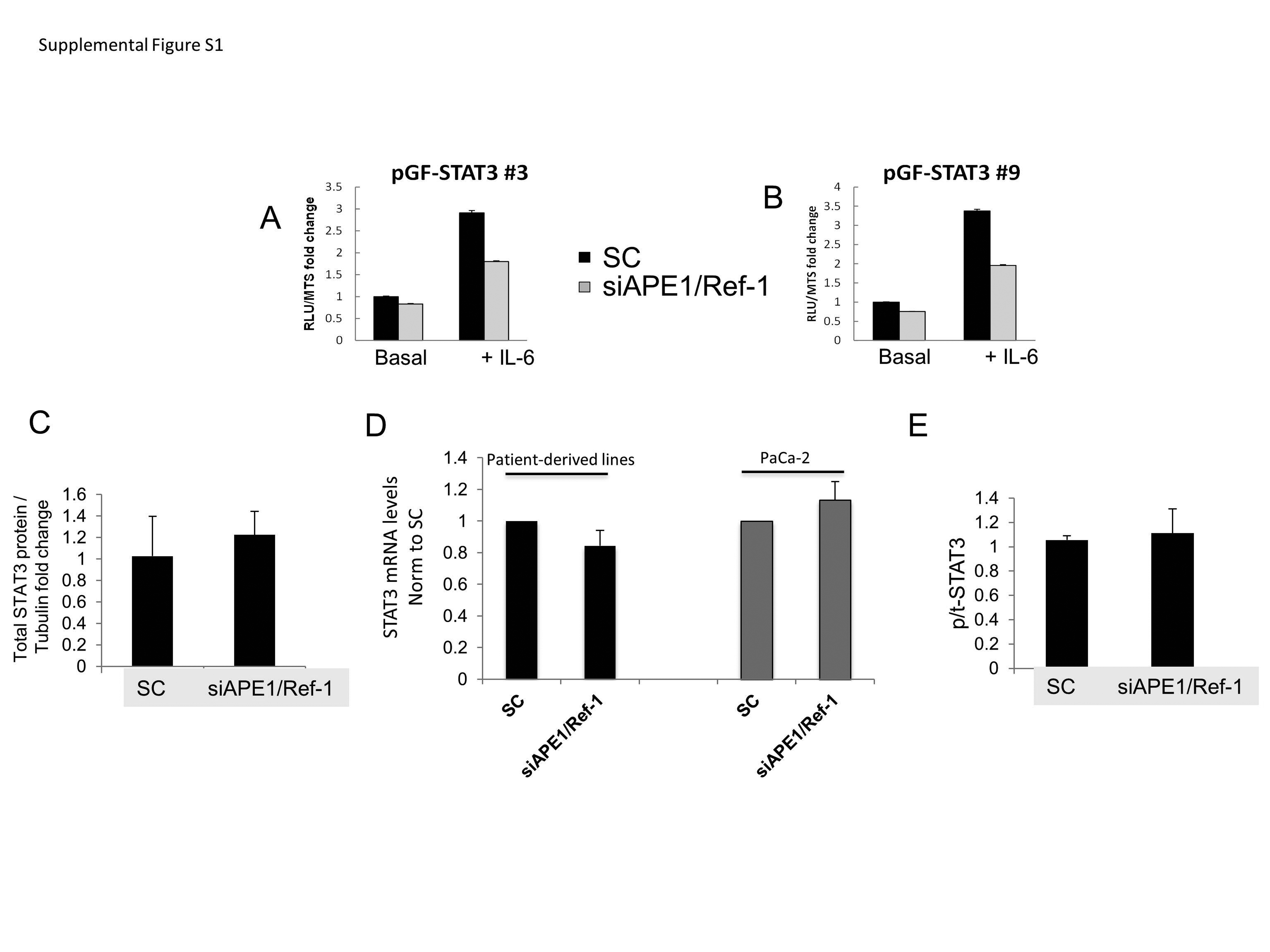

Supplement: Figure S1 — STAT3 activity is inhibited by APE1 knockdown, however STAT3 mRNA and protein levels do not change. Representative experiment of Panc-1 cells transduced with pGF-STAT3-Luc clones #3 (A) and #9 (B) following transfection with scrambled or APE1 siRNA (50 nM) and induced with IL-6 (50 ng/mL, 6 hr). C) Quantitation of Western blot of total STAT3 protein levels after APE1/Ref-1 knockdown in PaCa-2 cells. Total STAT3 levels were normalized to Tubulin. D) The amount of mRNA for STAT3 was analyzed by qPCR, using RPLP0 as the internal control for patient-derived lines (black bars) and Actin mRNA as the internal control for PaCa-2 (gray bars). For the patient-derived lines, the mRNA from three specimens was measured separately, in triplicate, and then averaged. PaCa-2 was done in three separate experiments in triplicate and the data averaged. E) Quantitation of Western blot for p-STAT3 levels following APE1 knockdown in PaCa-2 cells. p-STAT3 levels were normalized to total STAT3. Data represent average ± SD and are expressed as treated to scrambled (SC) control (n = 4–6). (TIF) [file pone.0047462.s001.tif]

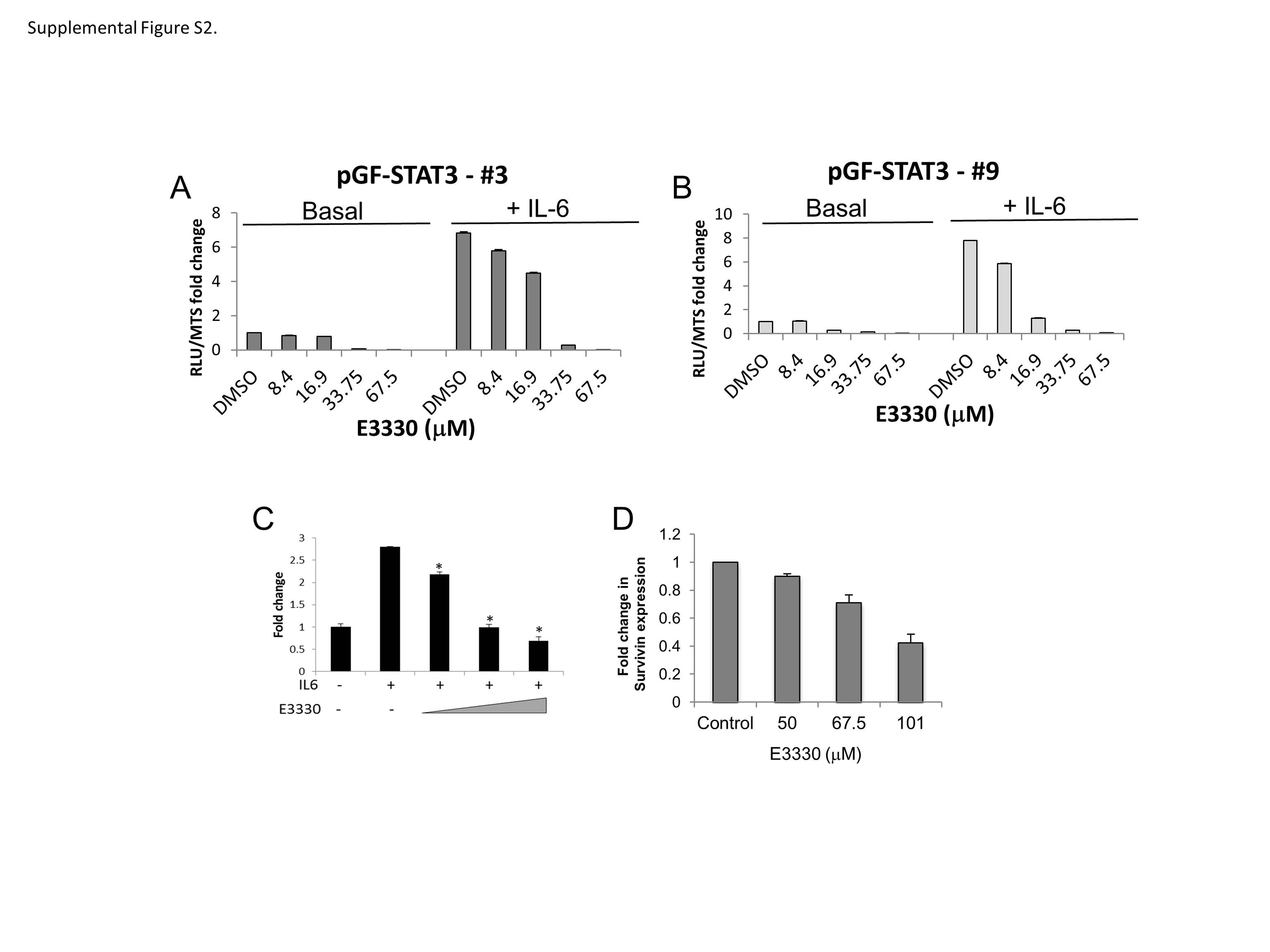

Supplement: Figure S2 — Inhibition of APE1 redox activity results in a decrease in STAT3 activity via reporter assay and target gene expression. A, B) Representative experiment of STAT3 activity following treatment of Panc-1 clones with APE1 redox inhibitor, E3330. C) Panc-1 cells were transiently transfected with STAT3-Luc construct and cotransfected with a Renilla vector, pRL-TK. After 16 h, cells were treated with E3330 for 24 h, IL6 (50 ng/mL) for 6 h, and Firefly and Renilla luciferase activities were assayed using Renilla luciferase activity for normalization. All transfection experiments were performed in triplicate and repeated at least 4 times in independent experiments. Data are expressed as Relative Luciferase Units (RLU) normalized to DMSO, and mean ± SE are shown. Student’s t tests were performed; * p<0.05, comparing E3330 versus DMSO. D) Expression of STAT3 target gene, survivin goes down following E3330 treatment (24 hr) in PaCa-2 cells (n = 3, avg±SD) via qPCR. (TIF) [file pone.0047462.s002.tif]

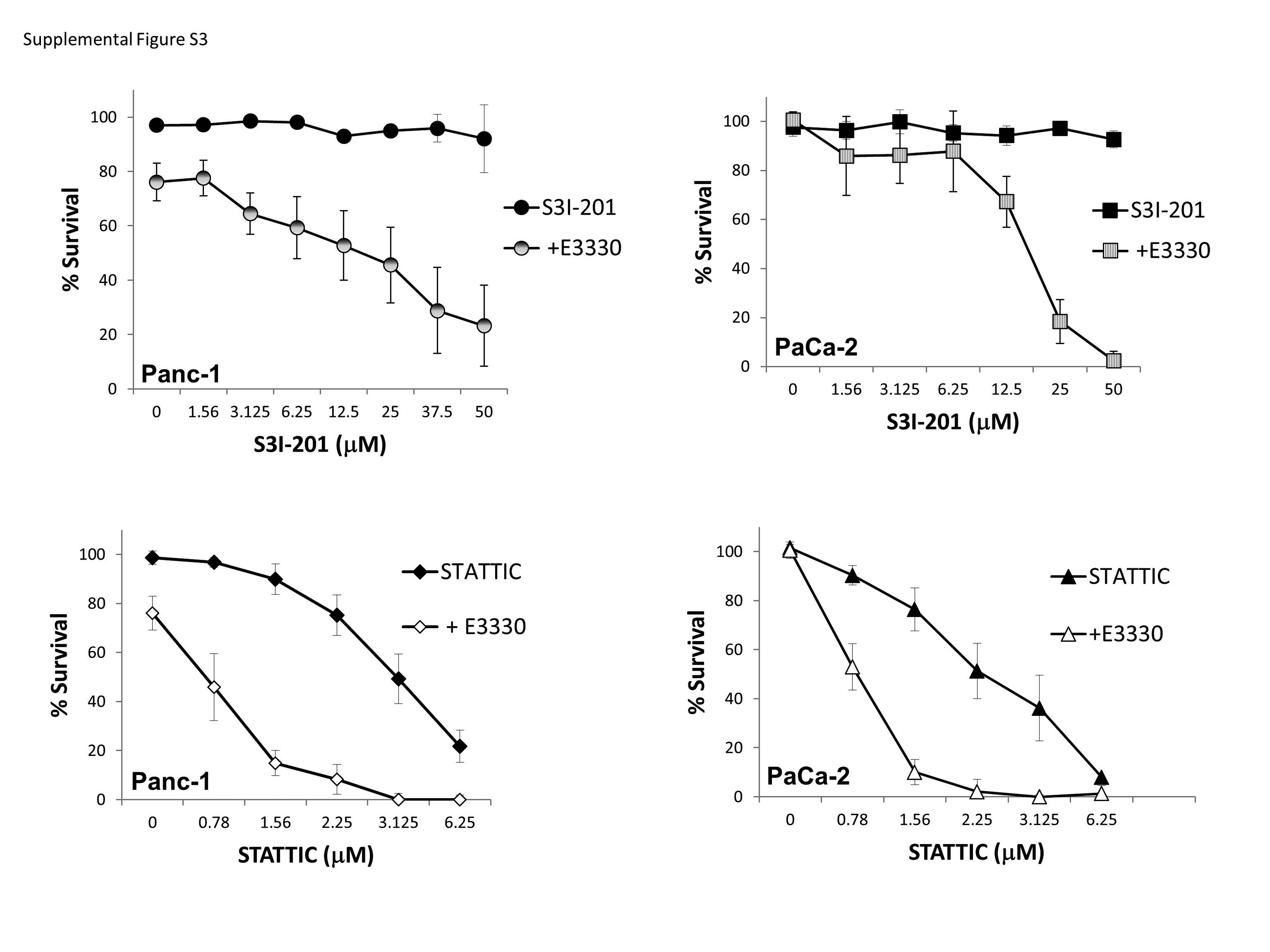

Supplement: Figure S3 — STAT3-APE1 dual targeting effectively inhibits PDAC cell proliferation. MTS assay was used to determine cell survival. Both drugs were added and were present for 72 h. Panc-1 and PaCa-2 were treated with 50 µM E3330. DMSO was tested as vehicle control. Data shown as mean ± SE of at least four independent experiments. (TIF) [file pone.0047462.s003.tif]

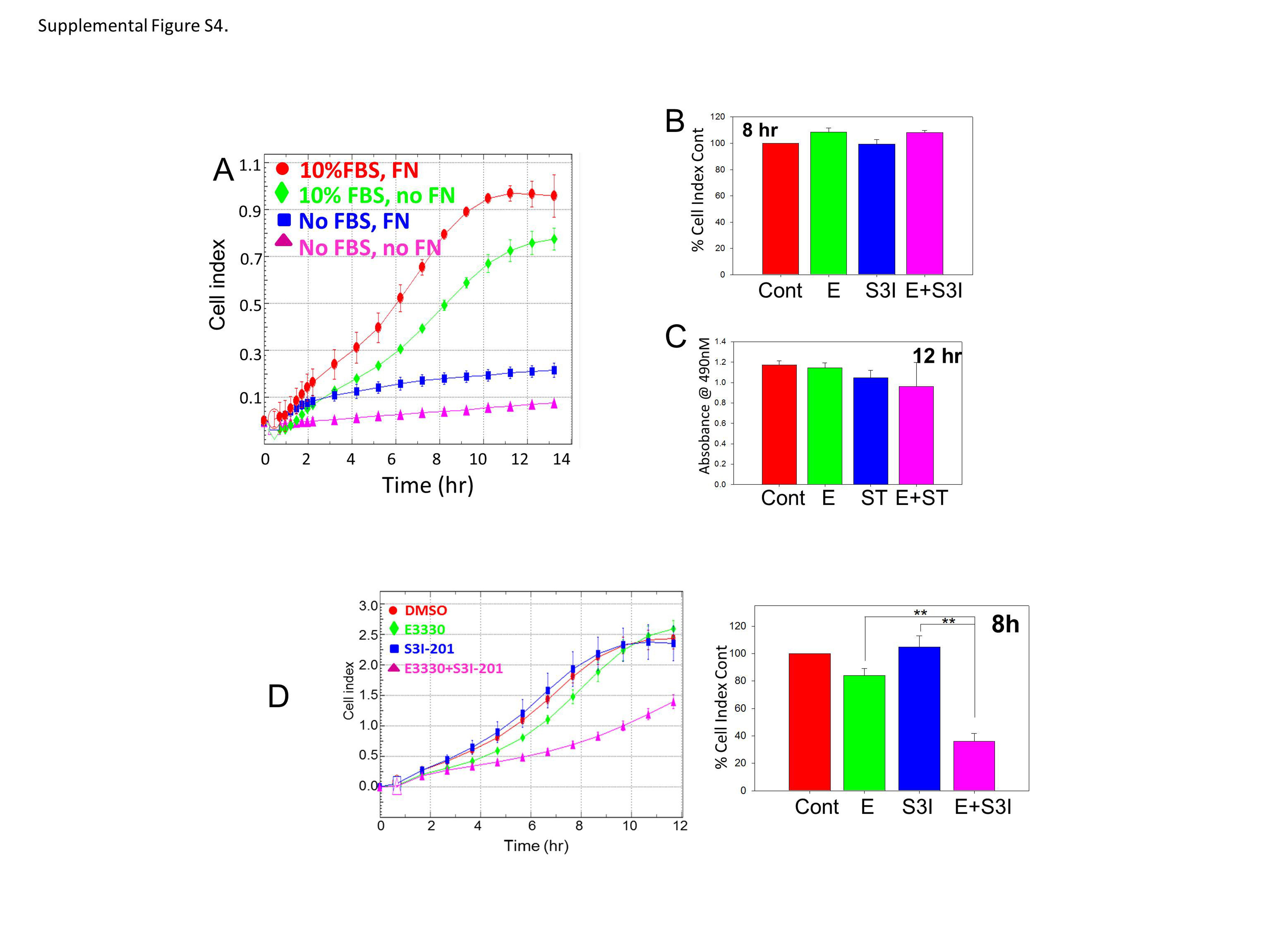

Supplement: Figure S4 — Combination of STAT3 and APE1 inhibitors inhibit PDAC cell migration. A) Panc-1 cells were serum-starved overnight. Cells (6×10^5^) were plated in duplicate in the upper chamber CIM plates with or without FN coating. Cells were also plated in the presence and absence of FBS in the lower chamber. Readings were taken for 12 h following plating. To demonstrate that the cells plated for migration shown in Figure 7 were indeed viable and that the migration of live cells was being monitored, we tested concurrent E-plate assays (B) using the xCELLigence system or MTS assays (C). Cont = DMSO, E = E3330, S3I = S3I-201, ST = STATTIC. D) Treatment with E3330 (75 µM) with STAT3 inhibitor S3I-201 (100 µM), dramatically reduces the cells’ migratory ability. Quantitation of three individual experiments at 8 hr is shown in F and G. ** p<0.01 using paired t test comparing S3I-201 alone with combination treatment. (TIF) [file pone.0047462.s004.tif]

## Slide 1
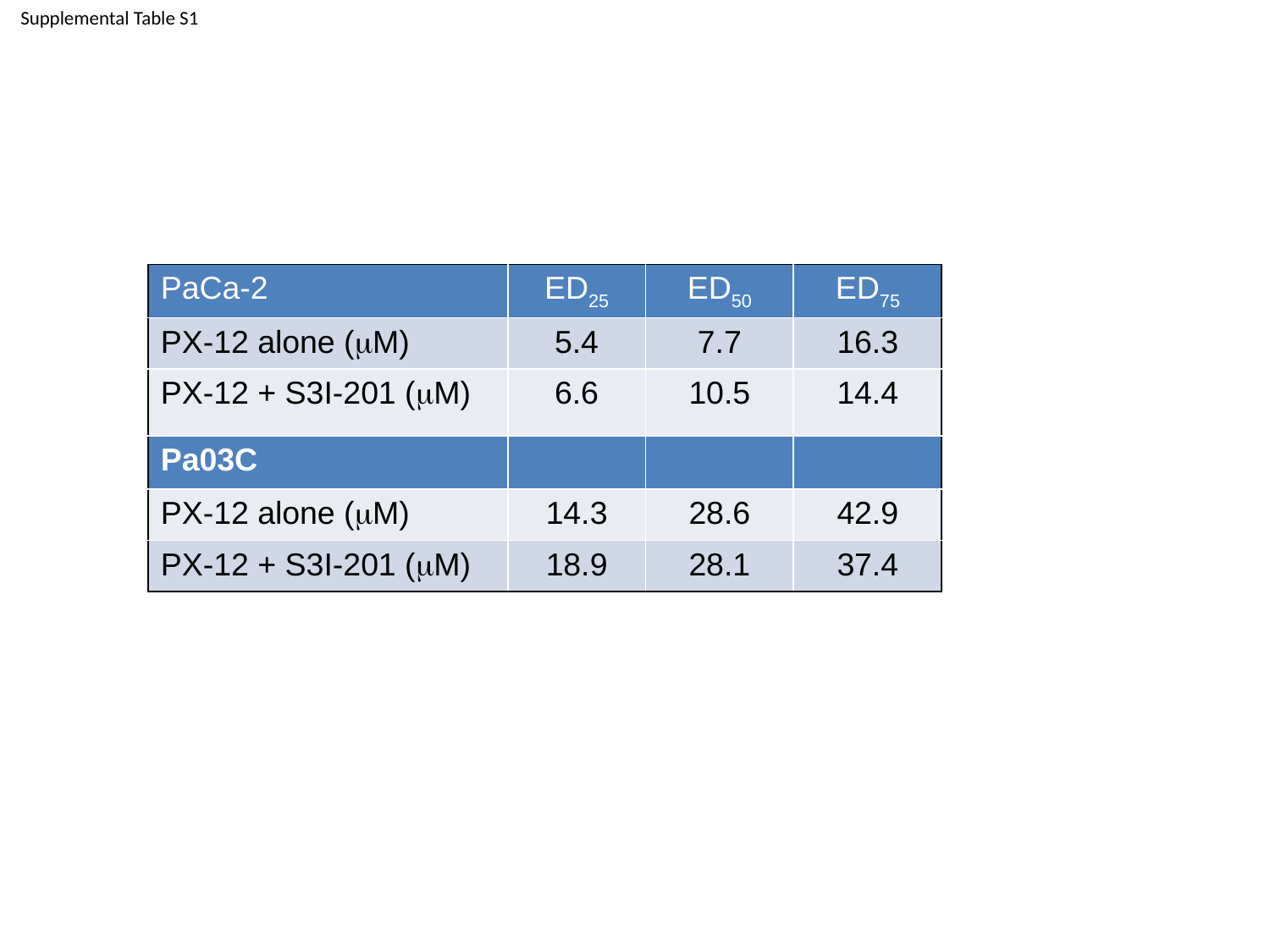

Supplemental Table S1
| PaCa-2 | ED25 | ED50 | ED75 |
| --- | --- | --- | --- |
| PX-12 alone (mM) | 5.4 | 7.7 | 16.3 |
| PX-12 + S3I-201 (mM) | 6.6 | 10.5 | 14.4 |
| Pa03C | | | |
| PX-12 alone (mM) | 14.3 | 28.6 | 42.9 |
| PX-12 + S3I-201 (mM) | 18.9 | 28.1 | 37.4 |

Supplement: Table S1 — Dual targeting of thioredoxin and STAT3 is not synergistic in PDAC cells. ED25, −50, and −75′s were determined using the MTS assay. (PPTX) [file pone.0047462.s005.pptx]
